# Proteomic Analysis Reveals That Iron Availability Alters the Metabolic Status of the Pathogenic Fungus *Paracoccidioides brasiliensis*

**DOI:** 10.1371/journal.pone.0022810

**Published:** 2011-07-28

**Authors:** Ana F. A. Parente, Alexandre M. Bailão, Clayton L. Borges, Juliana A. Parente, Adriana D. Magalhães, Carlos A. O. Ricart, Célia M. A. Soares

**Affiliations:** 1 Laboratório de Biologia Molecular, Instituto de Ciências Biológicas, Universidade Federal de Goiás, Goiânia, Goiás, Brazil; 2 Laboratório de Química de Proteínas, Departamento de Biologia Celular, Universidade de Brasília, Brasília, Distrito Federal, Brazil; Cinvestav, Mexico

## Abstract

*Paracoccidioides brasiliensis* is a thermodimorphic fungus and the causative agent of paracoccidioidomycosis (PCM). The ability of *P. brasiliensis* to uptake nutrients is fundamental for growth, but a reduction in the availability of iron and other nutrients is a host defense mechanism many pathogenic fungi must overcome. Thus, fungal mechanisms that scavenge iron from host may contribute to *P. brasiliensis* virulence. In order to better understand how *P. brasiliensis* adapts to iron starvation in the host we compared the two-dimensional (2D) gel protein profile of yeast cells during iron starvation to that of iron rich condition. Protein spots were selected for comparative analysis based on the protein staining intensity as determined by image analysis. A total of 1752 protein spots were selected for comparison, and a total of 274 out of the 1752 protein spots were determined to have changed significantly in abundance due to iron depletion. Ninety six of the 274 proteins were grouped into the following functional categories; energy, metabolism, cell rescue, virulence, cell cycle, protein synthesis, protein fate, transcription, cellular communication, and cell fate. A correlation between protein and transcript levels was also discovered using quantitative RT-PCR analysis from RNA obtained from *P. brasiliensis* under iron restricting conditions and from yeast cells isolated from infected mouse spleens. In addition, western blot analysis and enzyme activity assays validated the differential regulation of proteins identified by 2-D gel analysis. We observed an increase in glycolytic pathway protein regulation while tricarboxylic acid cycle, glyoxylate and methylcitrate cycles, and electron transport chain proteins decreased in abundance under iron limiting conditions. These data suggest a remodeling of *P. brasiliensis* metabolism by prioritizing iron independent pathways.

## Introduction

Iron is an essential nutrient for cellular function, but iron overload can be as detrimental as iron depletion. Thus, microorganisms use a complex network of systems to control iron levels in order to prevent free radical damage to proteins, ribonucleic acids, and cell membranes, keeping iron unavailable [1]. The absence of free iron in host tissues and host iron restriction mechanisms demand that pathogens develop an efficient iron uptake system in order to compete with the host for iron [2].

Iron deprivation responses in several fungi have been studied by transcriptional and proteomic analyses [3], [4], [5]. Iron homeostasis mechanisms including iron uptake, storage, and regulation have been extensively characterized especially in the fungal prototype *Saccharomyces cerevisiae*. Iron levels in *S. cerevisiae* are controlled by two major transcriptional factors identified as Aft1p and Aft2p. These two orthologues are missing in the majority of fungal species [6], [7], [8].

The human fungal pathogen A*spergillus fumigatus* also has a well characterized iron acquisition system. Under iron starvation conditions *A. fumigatus* employs two high affinity iron uptake systems that include siderophore-assisted and reductive iron uptake [9]. *A. fumigatus* iron acquisition under iron-depleted conditions is controlled by transcriptional factors encoded by the genes s*reA* and *hapX* [10], [11]. Under low iron conditions, the expression of the GATA factor SreA is reduced resulting in desrepression of the bZip transcription factor HapX. Genes involved in reductive iron assimilation, siderophore biosynthesis and uptake are also affected [11]. This regulatory circuit is largely conserved and orthologs to *sreA* and *hapX* are found in most fungal species [10], [12]. Moreover, the *A. fumigatus* zinc cluster transcription factor AcuM suppresses *sreA* and induces *hapX* to stimulate expression of genes involved in reductive and siderophore mediated iron uptake [13].

*Paracoccidoides brasiliensis* is a dimorphic fungus and the etiologic agent of paracoccidioidomycosis (PCM). The disease is restricted to Latin America, and is the most prevalent systemic mycosis in the region [14]. PCM is a major public health concern in rural areas. Moreover, this infection can lead to potentially disabling injuries. Several studies indicate that the majority of PCM cases are reported in Brazil [15] with an annual mortality rate of 148 deaths per year [16].

Searching analyses on *P. brasiliensis* genome database (http://www.broadinstitute.org/annotation/genome/paracoccidioides_brasiliensis/MultiHome.html) showed that this fungus possess genes that encode proteins with sequence similarity to host iron uptake systems which utilize heme while also employing siderophore-assisted iron uptake and reductive iron assimilation [17]. The fungal genome of *P. brasiliensis* contains sequences that potentially encode all the necessary enzymes for siderophore biosynthesis; *sidA*, *sidF*, *sidC* and s*idD* orthologs of *A. fumigatus* [17]. Putative *P. brasiliensis* siderophore transporters were identified as orthologs to genes encoding the *S. cerevisiae* Sit1p and the *A. nidulans* MirB and MirC [17]. Further investigation of the *P. brasiliensis* genome revealed that it contains orthologs to *hapx* and *sreA*. Despite these advances the identity and total number of genes and proteins involved in *P. brasiliensis* response to iron deprivation remains unclear.

Different reports indicate that iron depletion in fungi promotes the metabolic remodeling of iron-dependent processes including oxidative respiration, amino acid biosynthesis, and fatty acid metabolism. [3], [4], [18]–[20]. Altered expression of heat shock proteins has also been found in fungi during iron limiting conditions most likely due to the accumulation of unfolded proteins in the cytoplasm [21].

In this study we utilized 2D gel electrophoresis coupled to mass spectrometry to identify *P. brasiliensis* proteins sensitive to low iron levels. We discovered 274 differentially regulated proteins and 96 of these were identified, rendering an integrated view of metabolic and cellular processes reorganization during iron deprivation. Moreover, *in vivo* analysis confirmed the expression pattern of selected genes. These data suggest that the remodeling of *P. brasiliensis* metabolism is one way the fungus adapts and survives in a nutrient deficient environment.

## Results

### Iron overload exacerbates *P. brasiliensis* infection in BALB/c mice

**Figure 1:**
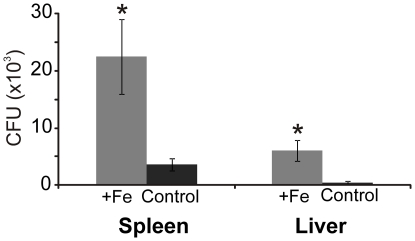
Iron supplementation increases mice susceptibility to *P. brasiliensis* infection. Colony forming units recovered from the spleen and liver of mice infected with *P. brasiliensis*. Mice were supplemented with iron (grey bars) or 0.9% (w/v) NaCl (black) for 15 days prior and during infection. Error bars represent standard deviation from four biological replicates while * represents *p*≤0.05.

To investigate the effect of iron availability on *P. brasiliensis* colonization we infected BALB/c mice that were iron supplemented. We observed an increased CFU recovery from liver and spleen of mice treated with an iron supplement (Figure 1). The results suggest that iron availability increases the susceptibility of mice to *P. brasiliensis* infection.

### Expression of *P. brasiliensis* iron acquisition genes during iron starvation

**Figure 2:**
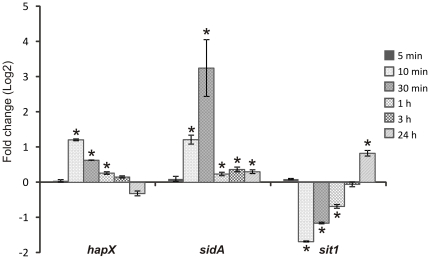
Induction of *P. brasiliensis* iron acquisition genes following iron restriction. Quantitative RT-PCR determined *P. brasiliensis* transcript levels of *hapX*, *sidA* and *sit1* during iron deprivation. Data were normalized to the L34 protein transcript. Student's t test was used for statistical comparisons. Error bars represent standard deviation from three biological replicates while * represents *p*≤0.05.

Before we began our proteomic analysis, we first sought to verify that our iron limiting conditions were restricting iron to the fungus by analyzing *P. brasiliensis* gene expression. To investigate the transcriptional profile of iron responsive genes in *P. brasiliensis* we used real-time RT-PCR. These genes included *P. brasiliensis* orthologs to the transcriptional regulator *hapX*, siderophore transporter *sit1*, and the siderophore biosynthesis gene *sidA*, encoding L-ornithine N^5^ –monooxygenase (Figure 2 and Table S1). In the first 10 minutes of iron deprivation, we observed an increased expression of the *hapX* and *sidA* orthologs. The level of *hapX* increased 2.3 times at 10 minutes of iron deprivation and the transcript level remained increased up to 1 hour in this condition. The *sidA* ortholog presented an expression level increased nine times compared to control following 30 minutes of iron starvation; transcript level remained at significant high levels in 24 hours of iron deprivation. The *sit1* ortholog was over expressed following 24 hours of iron deprivation. These results indicate that *P. brasiliensis* was subjected to iron restriction conditions because the known siderophore iron uptake system is active upon iron restriction.

### *P. brasiliensis* cell viability

Because we did not want to measure a proteome response which would represent cell death, we next sought to determine whether iron deprivation influenced *P. brasiliensis* cell viability. Using trypan blue staining we observed no significant differences in yeast cell viability up to 48 hours in iron deprivation (Figure S1). We did not observe a decrease in *P. brasiliensis* cell viability until 72 hours of exposure to iron limiting conditions (data not shown). These data show that the iron limiting incubation times selected for proteomic analysis did not influence *P. brasiliensis* cell viability.

### 2D-gel analysis of *P. brasiliensis* during iron starvation

**Figure 3:**
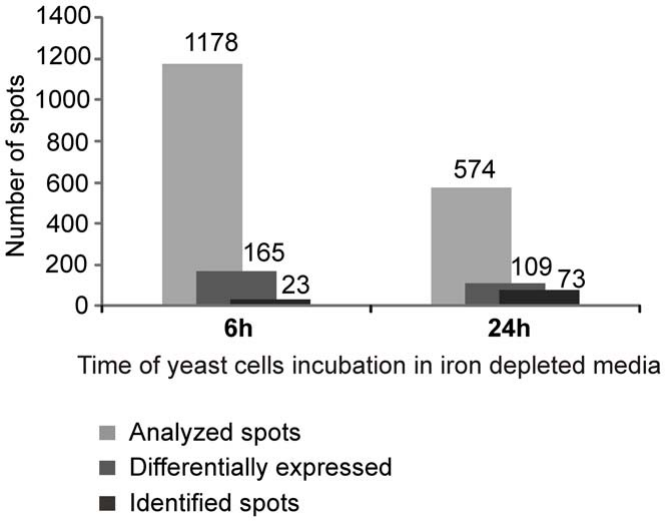
Graphic summation of *P. brasiliensis* iron restriction proteomic analysis. Number of spots differentially expressed were determined using 2D-gel image analysis software. Statistical analysis of the matched spots were performed using ANOVA.

**Figure 4:**
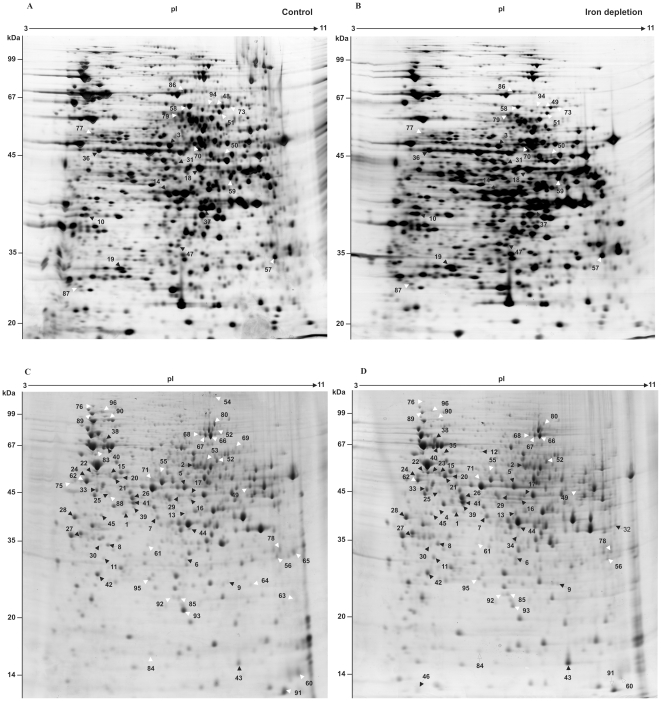
*P. brasiliensis* protein regulation during iron limiting conditions detected using 2D-gel analysis. 2D-gel analysis of *P. brasiliensis* proteins extracted from yeast cells grown in iron depleted media for 6 hours (B) and 24 hours (D). Gels A and C represent iron rich conditions. Black and white arrows indicate up-regulated and down-regulated proteins respectively. Identified protein spots are numbered and listed in Tables 1 and 2.

**Figure 5:**
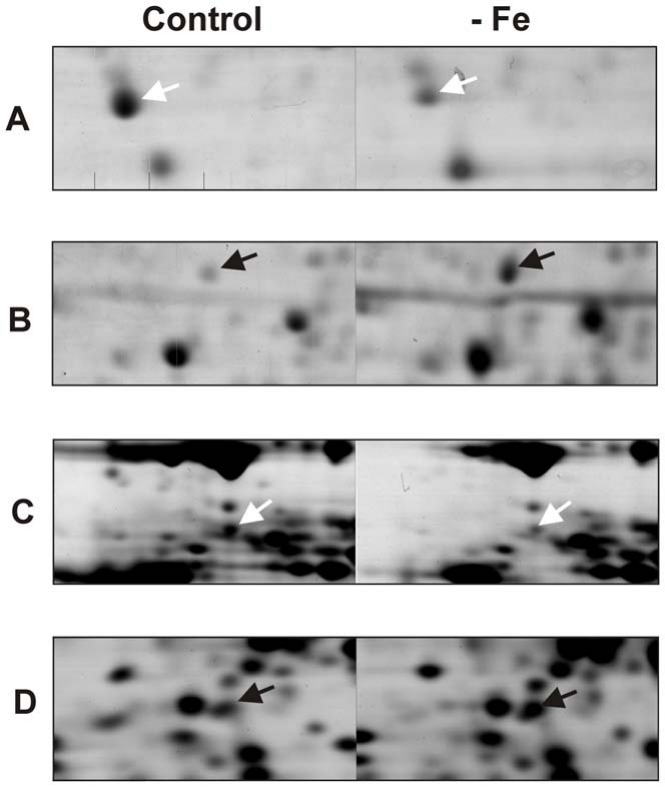
Magnified regions of the protein spots exhibiting protein abundance changes in the 2D gels shown in **Figure 4**. Protein spots correspond to the following proteins identified by mass spectrometry; Y20 protein (A), ATP synthase subunit beta (B), Tubulin alpha-1 chain (C), Conserved hypothetical protein (D). Black and white arrows indicate up-regulated and down-regulated proteins respectively.

Based on the results described above 6 and 24 hours iron depletion incubation times were selected for proteomic analysis of *P. brasiliensis* yeast cells. Two-dimensional gel analysis was used to separate cytosolic fungal proteins while image analysis allowed for the quantification of proteins. Three independent experiments generate three replicates which included: 6 hours in iron depletion, 6 hours control, 24 hours in iron depletion, and 24 hours control. Using the gel image software a total of 1752 spots were successfully matched between control and iron depletion conditions (Figure 3 and Figure 4). Statistical analysis revealed a difference in protein abundance for 165 and 109 protein spots following 6 and 24 hours of iron deprivation respectively. This yielded a total of 274 differentially regulated proteins (Figure 3). A magnified gel region allows for visualization of representative spots with differential abundance (Figure 5).

### Identification iron-regulated proteins

In order to determine the identities of the protein spots determined to be differentially regulated following 2D-gel analysis protein spots were subjected to in-gel digestion using trypsin and analyzed by mass spectrometry (MS). MS analysis following by protein database sequence matching resulted in the identification of 96 differentially expressed proteins (Figures 3 and 4). Seventy three proteins/isoforms were identified by peptide mass fingerprinting (PMF) and confirmed by MS/MS analysis while 23 of protein spots yielded identification by PMF only.

Among the 96 identified proteins 49 increased in abundance and 47 decreased. The *p*-values associated with each spot comparison are listed in Tables S2 and S3. Differences in protein spot intensity ranged from 1.2 to 3.6 (Tables 1 and 2). GenBank general information identifiers (gi), Broad Institute *P. brasiliensis* database accession numbers (PAAG), PMF and MS/MS mascot scores, protein molecular weights, and isoelectric points (*p*I) of each spot are also listed in Tables 1 and 2.

**Table 1:** *P. brasiliensis* identified proteins with increased expression during iron starvation.

Spot^1^	Time^2^	GenBank gi^3^	Protein identification	PMF score	MS/MS score	MW^4^	p*I* 5	≥fold change^6^
**METABOLISM**								
**Nucleotide metabolism**								
**1**	24 h	gi|226280544	Adenosine kinase	193	49	36.6/41.3	5.4/5.5	1.3
**2**	24 h	gi|226283962	GMP synthase	133	-	60.3/55.2	6.2/7.4	3.6
**C-compound and carbohydrate metabolism**								
**3**	6 h	gi|295664927	ATP-citrate-lyase	159	303	52.8/46.6	5.9/6.9	1.2
**Amino acid, nitrogen and sulfur metabolism**								
**4**	24 h	gi|226280080	2-nitropropane dioxygenase	163	107	38.8/41.9	5.4/5.1	**
**5**	24 h	gi|27368050	Formamidase	108	100	46.1/46.1	6.1/7.1	1.3
**6**	24 h	gi|226284927	Hydroxyacylglutathione hydrolase	66	86	28.9/33.1	6.1/7.1	1.7
**7**	24 h	gi|226282479	L-threonine 3-dehydrogenase	105	129	38.0/40.3	5.8/6.4	1.9
**8**	24 h	gi|226293104	Spermidine synthase	93	32	33.6/36.0	5.3/5.1	1.6
**Lipid, fatty-acid and isoprenoid metabolism**								
**9**	24 h	gi|226279101	Carbonic anhydrase	90	104	32.6/27.4	9.1/8.2	1.8
**Phosphate metabolism**								
**10**	6 h	gi|295672504	Inorganic pyrophosphatase	141	67	33.3/38.0	5.1/4.8	1.3
**ENERGY**								
**Electron transport and membrane-associated energy conservation**								
**11**	24 h	gi|226282053	ATP synthase subunit beta	199	118	55.1/33.3	5.2/5.0	2.5
**Glycolysis and gluconeogenesis**								
**12**	24 h	gi|146762537	Enolase	110	173	47.4/63.4	5.6/6.2	**
**13**	24 h	gi|29826036	Fructose 1,6-biphosphate aldolase	163	174	39.7/41.1	6.1/7.1	2.6
**14**	6 h	PAAG_01995	Fructose 1,6-biphosphate aldolase	77	-	39.6/40.3	6.0/6.8	1.4
**15**	24 h	gi|226279559	Glucokinase	113	49	55.7/52.2	5.3/5.0	2.8
**16**	24 h	gi|226285327	Phosphoglycerate kinase	108	72	45.3/43.4	6.4/7.3	1.6
**17**	24 h	gi|295669690	Phosphoglycerate kinase	149	72	45.3/44.1	6.4/7.3	1.8
**18**	6 h	gi|295669690	Phosphoglycerate kinase	82	53	45.3/42.9	7.8/7.7	1.2
**19**	6 h	gi|295670663	Triosephosphate isomerase (Tpi)	185	87	27.1/30.3	5.3/5.6	1.4
**Tricarboxylic-acid pathway**								
**20**	24 h	gi|295673931	Pyruvate dehydrogenase protein X component	110	41	52.7/50.6	6.4/5.4	1.5
**21**	24 h	gi|226280161	Pyruvate dehydrogenase protein X component	100	131	52.7/50.3	6.4/5.3	1.4
**CELL CYCLE AND DNA PROCESSING**								
**22**	24 h	gi|225683196	Tubulin alpha-2 chain	105	-	50.5/52.4	5.0/4.7	1.2
**23**	24 h	gi|225683196	Tubulin alpha-2 chain	117	57	50.5/53.2	5.0/4.9	**
**24**	24 h	gi|226285902	Tubulin beta chain	146	65	50.3/51.1	4.8/4.5	1.8
**25**	24 h	gi|154705473	Septin-1	123	87	44.1/44.3	5.2/5.1	2.0
**26**	24 h	gi|226294796	Actin	78	59	38.1/43.5	7.1/5.7	1.6
**27**	24 h	gi|38569374	14-3-3-like protein 2	120	84	29.7/37.1	4.6/4.4	1.7
**28**	24 h	gi|226282286	DNA damage protein rad24 (14-3-3 protein)	147	252	32.4/40.1	4.7/4.2	1.4
**TRANSCRIPTION**								
**29**	24 h	gi|226280907	mRNA binding post-transcriptional regulator (Csx1)	92	44	42.6/44.2	6.3/6.9	2.0
**30**	24 h	gi|226284577	Type 2A phosphatase activator tip41	90	-	35.9/27.9	5.3/4.9	2.3
**31**	6 h	PAAG_06891	mRNA binding post-transcriptional regulator (Csx1)	96	-	42.4/43.4	6.3/7.1	2.0
**PROTEIN SYNTHESIS**								
**32**	24 h	gi|226280659	60S ribosomal protein L5	104	-	34.3/39.7	9.0/9.7	**
**33**	24 h	gi|226282202	ATP-dependent RNA helicase eIF4a	130	60	45.0/45.1	5.1/4.8	1.3
**34**	24 h	gi|226280705	Elongation factor 2	103	110	92.6/37.8	6.4/7.2	**
**35**	24 h	gi|226283670	Translation initiation factor eIF3	84	56	66.0/62.3	5.1/5.2	**
**PROTEIN FATE (folding, modification, destination)**								
**36**	6 h	PAAG_05417	Mitochondrial-processing peptidase	140	-	53.0/45.2	5.8/7.1	1.3
**37**	6 h	gi|295661059	G-protein complex beta subunit cpcb	102	228	35.4/38.5	6.5/7.9	3.3
**CELL RESCUE, DEFENSE AND VIRULENCE**								
**38**	24 h	gi|60656557	Heat shock protein 90	148	91	80.3/73.7	4.9/5.0	1.4
**39**	24 h	gi|226285144	Hsp90 co-chaperone AHA1	150	48	36.5/42.0	5.4/5.7	1.4
**40**	24 h	gi|31324921	Heat shock protein SSC1 (70 kDa)	135	225	73.8/67.1	5.9/5.1	1.5
**41**	24 h	gi|295659116	Hsp70-like protein	244	126	70.9/42.7	5.0/5.7	1.6
**42**	24 h	gi|226286087	Mitochondrial peroxiredoxin PRX1	197	97	24.8/29.2	5.2/4.8	1.2
**43**	24 h	gi|17980998	Y20 protein	92	82	21.6/15.5	6.0/8.5	2.2
**UNCLASSIFIED PROTEINS**								
**44**	24 h	gi|226278304	Conserved hypothetical protein	103	40	38.0/38.5	6.0/7.2	1.4
**45**	24 h	gi|226286114	Conserved hypothetical protein	92	114	34.7/40.6	5.3/4.9	1.6
**46**	24 h	gi|226279849	Conserved hypothetical protein	86	75	11.8/13.0	5.0/4.3	**
**47**	6 h	PAAG_06617	Conserved hypothetical protein	113	-	30.5/34.9	6.2/7.1	1.3

**Spots visualized only in iron-depleted condition;

1 Spots numbers refers to Figure 3;

2 Time in iron starvation condition;

3 GenBank general information identifier;

4 Molecular weight (theoretical/experimental);

5 p*I* (theoretical/experimental).

6 ≥fold in iron depletion.

**Table 2:** *P. brasiliensis* identified proteins with reduced expression during iron starvation.

Spot^1^	Time^2^	GenBank gi^3^	Protein identification	PMF score	MS/MS score	MW^4^	p*I* 5	≤fold change^6^
**METABOLISM**								
**Nucleotide metabolism**								
**48**	6 h	gi|295672652	Bifunctional purine biosynthesis protein ADE17	186	52	66.7/61.6	6.7/8.1	2.0
**C-compound and carbohydrate metabolism**								
**49**	24 h	gi|226277934	2-methylcitrate synthase (2-Mcs)	254	153	51.5/46.2	9.0/8.8	3.1
**50**	6 h	PAAG_04550	2-methylcitrate synthase (2-Mcs)	96	-	51.4/44.5	9.0/8.3	2.1
**51**	6 h	gi|295666177	Mitochondrial 2-methylisocitrate lyase	140	55	67.2/59.1	8.7/8.2	1.5
**52**	24 h	gi|116561512	Isocitrate lyase (Icl)	301	95	60.1/58.6	6.7/7.9	1.3
**53**	24 h	gi|116561512	Isocitrate lyase (Icl)	233	47	60.1/57.8	6.7/7.7	**
**Amino acid, nitrogen and sulfur metabolism**								
**54**	24 h	gi|226281118	Pentafunctional AROM polypeptide	189	-	166/122	6.3/7.9	**
**55**	24 h	gi|226277980	Acetamidase	177	-	59.2/52.7	5.8/6.4	1.6
**56**	24 h	gi|226285914	Adenylyl-sulfate kinase	172	41	23.8/33.5	8.6/9.5	1.9
**57**	6 h	gi|225679649	Adenylyl-sulfate kinase	125	55	23.8/32.7	9.1/9.7	1.1
**58**	6 h	gi|295663176	Sulfate adenylyltransferase	307	552	64.0/61.9	6.2/7.4	1.8
**59**	6 h	gi|295662426	Aspartate aminotransferase	121	137	50.8/41.8	8.3/8.5	1.6
**ENERGY**								
**Electron transport and membrane-associated energy conservation**								
**60**	24 h	gi|226279655	Cytochrome c	75	71	12.2/14.5	9.2/10.1	2.6
**61**	24 h	gi|225677786	ATP synthase gamma chain	87	-	32.4/36.0	6.7/6.1	1.6
**62**	24 h	gi|226282053	ATP synthase subunit beta	249	376	55.1/48.3	5.2/4.5	1.5
**63**	24 h	gi|226279593	ATP synthase subunit 5	112	76	24.6/23.5	9.7/10.1	**
**64**	24 h	gi|226291052	ATP synthase subunit 4	94	69	26.7/26.2	9.4/8.8	**
**65**	24 h	gi|226278316	Electron transfer flavoprotein subunit beta	103	136	21.6/34.3	9.3/10	**
**Tricarboxylic-acid pathway**								
**66**	24 h	gi|226293399	Aconitase	172	211	79.1/77.7	6.4/7.8	1.7
**67**	24 h	gi|225684009	Aconitase	146	184	85.0/79.0	7.2/7.7	1.7
**68**	24 h	gi|225684009	Aconitase	139	55	85.0/80.1	7.2/7.5	1.6
**69**	24 h	gi|226278535	ATP-citrate synthase subunit 1	84	-	72.4/65.9	8.0/8.4	**
**70**	6 h	PAAG_03330	Dihydrolipoyl dehydrogenase	86	-	55.6/47.0	8.2/7.6	1.3
**Oxidation of fatty acids**								
**71**	24 h	gi|226278634	Aldehyde dehydrogenase (Aldh)	217	197	54.5/49.7	5.8/6.2	2.2
**72**	24 h	gi|226286163	3-hydroxybutyryl CoA dehydrogenase	165	101	34.3/36.4	8.4/7.3	1.5
**73**	6 h	PAAG_06224	Carnitine O-acetyltransferase	89	-	69.4/61.7	8.2/8.5	2.1
**CELL CYCLE AND DNA PROCESSING**								
**74**	24 h	gi|154705473	Septin-1	151	105	44.1/44.3	5.2/5.1	**
**75**	24 h	gi|38569374	14-3-3-like protein 2	90	36	29.7/46.8	4.6/4.2	**
**76**	24 h	gi|226278903	Cell division cycle protein	311	114	90.5/118	4.9/4.7	3.5
**77**	6 h	PAAG_01647	Tubulin alpha-1 chain	103	-	50.0/50.7	5.0/5.0	1.7
**TRANSCRIPTION**								
**78**	24 h	gi|226277842	Prohibitin-1	180	-	30.9/35.1	8.7/9.6	1.6
**79**	6 h	gi|295673504	cwfJ domain-containing protein	88	-	61.6/58.3	6.1/7.2	1.4
**PROTEIN SYNTHESIS**								
**80**	24 h	gi|226280705	Elongation factor 2	283	244	92.6/92.2	6.4/7.9	3.0
**81**	24 h	gi|226280705	Elongation factor 2	189	-	92.6/85.0	6.4/7.9	**
**82**	24 h	gi|28395450	40S ribosomal S12 protein	95	63	16.8/19.3	5.0/4.4	1.7
**83**	24 h	gi|226282202	ATP-dependent RNA helicase eIF4a	142	63	45.0/64.1	5.1/4.8	**
**PROTEIN FATE (folding, modification, destination)**								
**84**	24 h	gi|226285231	Ubiquitin-conjugating enzyme variant MMS2	126	153	15.7/16.4	6.1/6.2	1.5
**85**	24 h	gi|34979129	Peptidyl-prolyl cis/trans isomerase	108	69	20.9/24.4	6.0/9.7	1.4
**86**	6 h	gi|295663176	Dipeptidyl-peptidase	201	84	86.2/70.0	7.9/7.3	1.7
**CELLULAR TRANSPORT AND TRANSPORT MECHANISMS**								
**87**	6 h	PAAG_03137	Vacuolar protein sorting-associated protein	72	-	22.1/26.3	5.0/4.6	1.2
**CELLULAR COMMUNICATION/SIGNAL TRANSDUCTION MECHANISM**								
**88**	24 h	gi|226285275	Stomatin family protein	89	-	48.7/43.4	6.5/5.1	**
**CELL RESCUE, DEFENSE AND VIRULENCE**								
**89**	24 h	gi|60656557	Heat shock protein 90	173	60	80.3/104	4.9/4.6	2.3
**90**	24 h	gi|31324921	Heat shock protein SSC1 (70 kDa)	214	263	73.8/96.8	5.9/5.1	2.0
**91**	24 h	gi|226278527	10 kDa heat shock protein, mitochondrial	161	108	11.1/12.9	8.7/9.7	1.4
**92**	24 h	gi|226282384	30 kDa heat shock protein	142	-	28.6/24.3	6.8/6.9	1.6
**93**	24 h	gi|17980998	Y20 protein	160	302	21.6/22.1	6.0/7.1	2.9
**94**	6 h	gi|24528587	Peroxissomal catalase	71	-	70.7/61.7	6.3/8.0	2.0
**UNCLASSIFIED PROTEINS**								
**95**	24 h	gi|226285365	Conserved hypothetical protein	135	194	26.0/28.2	5.8/6.1	1.2
**96**	24 h	gi|226286445	Conserved hypothetical protein	154	37	115/109	5.1/4.9	2.0

**Spots visualized only in iron-replete condition;

1 Spots numbers refers to Figure 3;

2 Time in iron starvation condition;

3 GenBank general information identifier;

4 Molecular weight kDa (theoretical/experimental);

5 p*I* (theoretical/experimental);

6 ≤fold in iron depletion.

Identified proteins were grouped together based on their functionality according to the Munich Information Center for Protein Sequences (MIPS). Pie charts representing the distribution of the 96 identified spots according to their biological function are shown in Figure S2. *P. brasiliensis* differentially regulated proteins were involved in several cellular processes including energy (26%), metabolism (22.9%), cell rescue and virulence (12.5%), cell cycle (11.5%), protein synthesis (10.4%), transcription (5.2%), protein fate (3.1%), cellular communication (1%) and cell transport (1%). Unclassified proteins represented only a small fraction (6.2%) of the 96 protein spots analyzed by MS.

Twenty six percent of the *P. brasiliensis* identified proteins were grouped into energy processes. Glycolytic enzymes including enolase, fructose 1,6-biphosphate aldolase, glucokinase, phosphoglycerate kinase and triosephosphate isomerase were up regulated ranging in fold change from 1.2 to 2.8. The increased regulation of glycolytic enzymes was observed at 6 and 24 hours of iron deprivation (Table 1). The abundance of hydroxyacylglutathione hydrolase was also increased correlating with the detected increase in glycolytic enzyme regulation because this enzyme plays role in detoxification of glycolysis products.

While the abundance of *P. brasiliensis* glycolytic pathway enzymes increased during iron deprivation the tricarboxylic acid cycle (TCA) enzymes were decreased. TCA enzyme included aconitase, one subunit of ATP-citrate synthase, and dihydrolipoyl dehydrogenase (Figure S2, Table 2) depicted a decreased abundance during iron starvation. Four subunits of the electron transport ATP-synthase complex also decreased in abundance during iron restriction. Two other proteins related to electron transport were also down-regulated in *P. brasiliensis* during iron deprivation. These proteins included cytochrome c and electron transfer flavoprotein beta-subunit.

**Figure 6:**
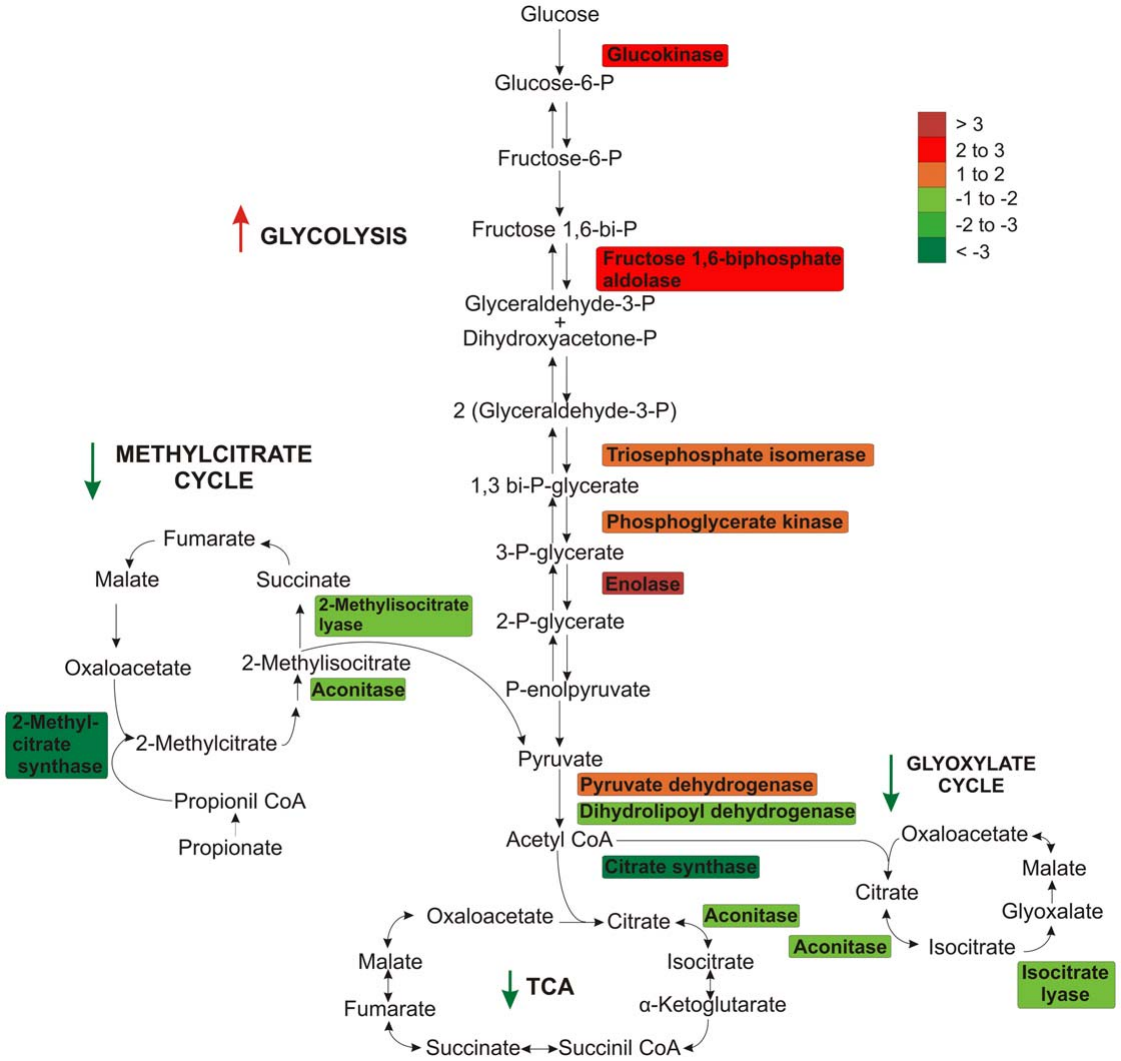
Overview of *P. brasiliensis* metabolic processes regulated during iron starvation revealed by proteomic analysis. Schematic representation of proteins involved in *P. brasiliensis* carbon metabolism (glycolysis/TCA cycle/glyoxylate cycle/methylcitrate cycle). Proteins are colored according to their differences in abundance as detected by 2D-gel analysis of *P. brasiliensis* following iron starvation. Arrows indicate up or down regulation of metabolic pathways.

*P. brasiliensis* proteins involved in pathways using alternative sources of carbon, such as glyoxylate and methylcitrate cycles were also decreased in abundance during iron starvation. Two isoforms of the glyoxylate cycle enzyme isocitrate lyase, were decreased in abundance after 24 hours in iron depletion (Figure S2, Table 2). These isoforms play a regulatory role in glyoxylate cycle allowing fungi to use fatty acids to synthesize carbohydrates. Two *P. brasiliensis* enzymes involved in the methylcitrate cycle were decreased in abundance following iron restriction. These enzymes were 2-methylisocitrate lyase and 2-methylcitrate synthase. Figure 6 summarizes the *P. brasiliensis* metabolic proteins regulated during iron starvation in this study.

We also detected a decreased regulation of *P. brasiliensis* enzymes related to fatty acids metabolism including aldehyde dehydrogenase, 3-hydroxybutyryl CoA dehydrogenase, and carnitine-O-acetyltransferase (Table 2). Enzymes related to amino acid metabolism were also decreased in abundance during iron starvation, such as the pentafunctional AROM polypeptide, related to aromatic amino acids biosynthesis, and aspartate aminotransferase, the enzyme responsible for the amination of oxaloacetate to form aspartate. In contrast L-threonine 3-dehydrogenase was 1.9 times fold higher in abundance following iron deficiency (Tables 1 and 2). This enzyme is involved in threonine catabolism which produces glycine.

Another *P. brasiliensis* cellular process affected by iron starvation was cell rescue. Eight proteins, including isoforms, belonging to the heat shock protein family were altered in abundance following iron limitation (Tables 1 and 2).

### *P. brasiliensis* mRNA and proteomic analysis correlate with in vivo infection

**Figure 7:**
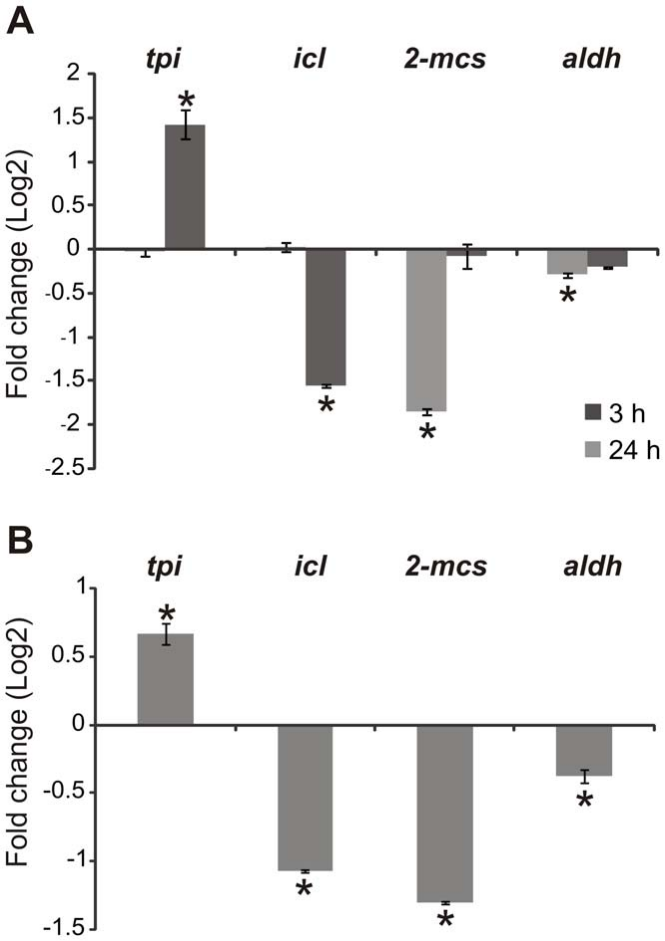
*P. brasiliensis* transcripts of yeast cells isolated from infected mice correspond to *P. brasiliensis* proteins differentially regulated under iron limiting conditions. *P. brasiliensis* transcript levels of genes encoding enzymes involved in energy processes; triosephosphate isomerase (*tpi*), isocitrate lyase (*icl*), 2-methylcitrate synthase (*2-mcs*) and aldehyde dehydrogenase (*aldh*), isolated from yeast cells following iron starvation (A), and from yeast cells recovered from the spleens of infected BALB/c mice (B). Transcript levels were measured using quantitative RT-PCR. Data were normalized to the L34 protein transcript and presented as log2 (fold change). Student's t test was used for statistical comparisons. Error bars represent standard deviation from three biological replicates while * represents *p*≤0.05.

In order to validate the significance of our proteomic results we next sought to determine if changes in protein levels could be correlated with changes in transcript levels *in vivo*. We determined that the differences observed in proteomic assay are in agreement with transcriptional findings, using quantitative RT-PCR to measure isocitrate lyase (*icl*), aldehyde dehydrogenase (*aldh*) and 2-methylcitrate synthase (*2-mcs*) transcripts (Figure 7A). Protein and transcript levels of these enzymes decreased during iron limitation as depicted in Figure 7A and Table 2. The triosephosphate isomerase (*tpi*) transcript level was correlated with protein levels (Figure 7A and Table 1). The same oligonucleotides were next used to evaluate the transcriptional levels of these genes in *P. brasiliensis* isolated from mouse spleens. We observed that the changes in transcriptional profile of the genes *tpi*, *icl*, *2-mcs* and *aldh* in yeast cells isolated from mice are similar to those observed in response to iron deprivation (Figure 7 B).

### Western blot analysis and enzymatic activity correlates with proteomic data

**Figure 8:**
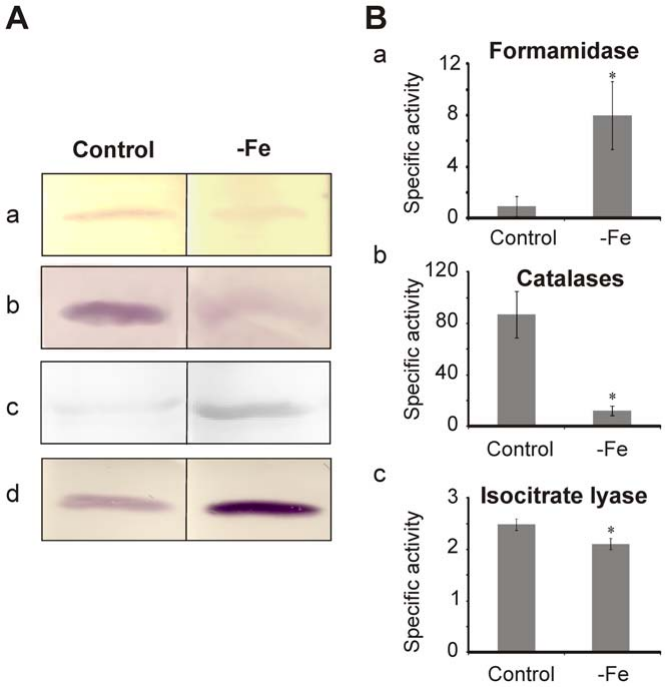
Western blot and enzymatic activity analysis validate proteomic data of *P. brasiliensis* yeast cells during iron-limiting condition. A) Western blot analysis of proteins probed with antibodies to (a) aconitase, (b) isocitrate lyase, (c) formamidase and (d) triosephosphate isomerase. B) Activity assay results from (a) formamidase, (b) catalases, (c) isocitrate lyase. Control represents yeast cells incubated for 24 hours in MMcM medium. −Fe represents yeast cells incubated for 24 hours in MMcM medium depleted of iron. Error bars represent standard deviation from three biological replicates while * represents *p*≤0.05.

Western blot analysis was performed to confirm the *P. brasiliensis* protein level changes detected in the proteomic analysis. This was accomplished using antibodies specific to four proteins affected by iron levels as determined by 2D gel analysis. *P. brasiliensis* aconitase and isoctrate lyase (Figure 8 A, panels a and b) levels following iron depletion were observed to decrease in abundance by western blot and 2D-gel analysis. Formamidase and triosephosphate isomerase also yielded similar increases in abundance following western blot analysis to that described by 2D-gel analysis (Figure 8 A panels c and d).

Moreover, we performed enzymatic assays for formamidase, catalases, and isocitrate lyase using *P. brasiliensis* protein extracts derived from yeast cells grown under iron deprivation in order further validate our proteomic data. We observed a statistically significant increase in formamidase activity (Figure 8 B, panel a) and a decrease in catalases and isocitrate lyase activity during iron starvation (Figure 8 B, panels b and c, respectively). All data again are in agreement with the 2D-gel analysis.

## Discussion

In this study, the treatment of mice with an iron supplement made them more susceptible to *P. brasiliensis* infection. Similar trends were also observed for the pathogenic fungi *C. neoformans* [22] and *A. fumigatus* [23]. Moreover, we found that *P. brasiliensis* regulated the abundance of 274 proteins following iron depletion. Host iron restriction is thought to be one part of the host defense mechanism. Our data suggest that a remodeling of *P. brasiliensis* metabolism by limiting the role of iron dependent pathways could be one mechanism in which the fungus adapts to the host environment.

Because iron is not abundantly available in host tissues pathogenic microorganisms have developed mechanisms which enable them to survive in low iron environments [24]. These mechanisms include iron uptake from host sources, and metabolic switching to iron independent pathways [25]–[31]. It is known that a component of the fungal cellular response to iron deficiency operates through the induction of a number of transcripts [18], [32]–[34]. Under our iron restriction conditions it was observed the induction of *P. brasiliensis* orthologs to the known iron dependent transcripts, such as the transcriptional regulator *hapX*, the siderophore biosynthetic enzyme *sidA* and the siderophore transporter *sit1* [17]. These results suggest the activation of the siderophore pathway of *P. brasiliensis* under iron limiting conditions. Moreover, the induction of *hapX* suggests that this ortholog in *P. brasiliensis* is also integral to the fungus response to iron starvation as previously described in other organisms [34]. These data support our *P. brasiliensis* iron restriction model for further analysis using proteomic technologies.

Proteomic analysis revealed that during iron deprivation the majority of *P. brasiliensis* regulated proteins were related to energy and metabolic function. Among the pathways potentially repressed in *P. brasiliensis* during iron depletion was the tricarboxylic acid cycle (TCA). TCA oxidative reactions are mediated by enzymes containing Fe/S clusters including aconitase, succinate dehydrogenase and fumarase [35]. We observed that TCA enzymes: aconitase, an ATP-citrate synthase subunit and dihydrolipoyl dehydrogenase, were regulated in *P. brasiliensis* by iron availability. These data taken together suggest metabolic switching to pathways independent of enzymes with Fe/S clusters. The decreased expression of TCA enzymes observed in *P. brasiliensis* during iron deprivation has also been reported in other pathogenic bacteria and fungi [3], [4], [20], [36], [37]. The evidence of TCA repression during iron deprivation is further emphasized by a decreased expression of the *P. brasiliensis* enzyme aspartate aminotransferase. A substrate for aspartate aminotransferase is the TCA intermediate metabolite oxaloacetate [38]. A reduction in the amount of this substrate due to TCA repression may influence the decreased expression of aspartate aminotransferase. The correlation between aspartate biosynthesis and TCA was also reported in mice given acetate. The authors of this study observed an increase in TCA flux concurrent with an increase in aspartate aminotransferase [39].

In addition to TCA cycle enzymes, a decrease in the abundance of *P. brasiliensis* glyoxylate cycle enzymes were also observed during iron depletion. The glyoxylate pathway provides cells with an alternative source of carbon allowing for the consumption of acetate and/or ethanol in place of glucose [40]. The reduced expression of enzymes involved in the glyoxylate pathway correlates with the decreased expression of TCA enzymes because these cycles are coupled. Two of the five reactions of the TCA cycle are catalyzed by enzymes unique to the glyoxylate cycle [41]. Reduced levels of isocitrate lyase, an enzyme with a key role in the glyoxylate cycle, correlate with the negative regulation of this pathway during iron deprivation. We could speculate that during iron limitation and with glucose as the carbon source, *P. brasiliensis* reduces the glyoxylate cycle activity due to the requirement of aconitase which is an iron-dependent enzyme of this pathway. This speculation is proven stronger because of the induction of *hapX*. This transcription factor negatively regulates iron dependent energy metabolism.

Two *P. brasiliensis* enzymes involved in the methylcitrate cycle were also decreased in abundance following iron restriction. The methylcitrate cycle is another system that provides an alternative source of carbon through pyruvate production [42]. Similar to the glyoxylate cycle this pathway functions using enzymes that are part of the TCA cycle [43]. These data add further support to the overall metabolic adjustment of *P. brasiliensis* under iron deprivation.

*P. brasiliensis* proteins involved in oxidative phosphorylation also decreased in abundance following iron limitation. Oxidative phosphorylation promotes ATP formation from NADH and FADH_2_ produced by the TCA cycle [44]. The suppression of pathways such as TCA that feed into oxidative phosphorylation may be associated with the decreased expression levels of enzymes that are part of oxidative phosphorylation. Moreover, *P. brasiliensis* enzyme complexes part of the electron transport chain utilize iron as a prosthetic group. Thus, the maintenance of this pathway is iron dependent and with iron levels being low their production may be down regulated as we have detected in this study.

Our data suggest that the suppression of oxidative pathways during iron deprivation in *P. brasiliensis* is linked to an increase of glycolytic activity as demonstrated by the induction of most enzymes in the glycolytic pathway. The glucose concentration of the medium for all of our proteomic analysis remained above 0.8% (w/v) providing high glucose availability to the yeast cells (data not shown). Increased glycolysis activity from iron limitation has been observed in other bacteria and fungi including *Staphylococcus aureus* and *S. cerevisiae* [20], [45]. Moreover, the increased expression of *P. brasiliensis* hydroxyglutatione hydrolase observed in this study is also linked to glycolysis induction. Hydroxyglutatione hydrolase is involved in methylglyoxal detoxification which is a toxic molecule generated from the degradation of glycolytic products [46]. Hydroxyglutatione hydrolase prevents methylglyoxal accumulation by converting it to D-lactate in presence of glutathione [47]. These results suggest that *P. brasiliensis* remodels its energy metabolism in response to iron starvation by increasing glycolytic activity to compensate for the decrease of aerobic pathways, that are iron dependent.

It is important to note that the abundance changes detected by proteomic analysis for enzymes involved in energy processes during iron depletion were also observed following transcriptional analysis of selected genes of *P. brasiliensis* isolated from mouse spleens. Similar to what has been observed for other bacterial and fungal pathogens, these data suggest that iron deprivation treatment may mimic the environment *P. brasiliensis* encounters in the host. To further support this theory, the induction of genes involved with glucose utilization under host conditions have been reported for other fungal pathogens. *C. albicans* over expresses *pfk2* that encodes for phosphofructokinase and *pyk1* that encodes for pyruvate kinase when growing within the kidney [48].

### Conclusions

Proteomic analysis of *P. brasiliensis* revealed that the major cellular response affected by iron restriction was related to energy production. Our data suggest that under iron limiting condition glycolysis was the more favored energy pathway over oxidative pathways that are dependent on enzymes containing Fe-S groups. Thus, *P. brasiliensis* relies more on the energy generated from glycolysis in order to compensate for the decrease in ATP production from oxidative phosphorylation. In addition to the activation of iron uptake systems, *P. brasiliensis* metabolic adjustment may be an indispensable survival mechanism the fungus requires when in a nutrient deficient environment.

## Materials and Methods

All animal experiments were performed in accordance with the international rules for animal experimentation. The animal protocol was approved by the Universidade Federal de Goiás committee of the ethical treatment of animals (Number: 131/2008).

### *P.brasiliensis* strain maintenance

*P. brasiliensis Pb* 01 (ATCC MYA-826) was used in all experiments. The yeast phase was maintained *in vitro* by sub culturing at 36°C in Fava Netto's semisolid medium [49] every 7 days. Fava Netto's semisolid medium components were as follows; 1% (w/v) peptone, 0.5% (w/v) yeast extract, 0.3% (w/v) proteose peptone, 0.5% (w/v) beef extract, 0.5% (w/v) NaCl, 4% (w/v) glucose, 1.2% (w/v) agar, pH 7.2.

### BALB/c mice infection

Thirty day old female BALB/c mice (*n* = 4) were injected intraperitoneally with a solution containing Ferrodex diluted in 0.9% NaCl (w/v; 50 mg/kg; Tortuga Companhia Zootécnica Agrária, Brazil). Ferrodex is a ferric hydroxide solution containing dextran commonly used for animal supplementation experiments. The iron source was given every 2 days for 15 days prior to and during infection. For control experiments (*n* = 4) sterile 0.9% NaCl (w/v) was injected into mice at the same time intervals as iron supplementation. Mice were inoculated intraperitonially with 10^7^ *P. brasiliensis* yeast cells as previously described [50]. After 2 weeks of harboring the organism, mouse spleens and livers were removed and homogenized in 5 mL of sterile 0.9% (w/v) NaCl. The homogenized sample was plated in brain heart infusion agar supplemented with 4% (v/v) fetal calf serum and 2% (w/v) glucose. The plates were incubated at 36°C and colony forming units (CFUs) were determined after 20 days.

Gene expression analyses of *P. brasiliensis* from infected mice were performed by isolating yeast cells from spleens as previously described with minor modifications [51]. The spleens of infected mice were homogenized in water using a grinder. To remove large pieces of animal tissue the homogenate was then filtered using a nylon membrane. The sample was frozen in liquid nitrogen and then centrifuged at 500× *g* for 5 minutes to remove any remaining animal tissue. Next, the sample was centrifuged at 7000× *g* for 15 minutes in order to isolate fungal cells. The samples containing fungal cells were checked under the microscope to make sure that they were not cross-contaminated by spleen cells. RNA was extracted from the fungal cells by using TRIzol reagent (Invitrogen, Carlsbad, CA, USA) following the manufacturer's protocol. It is important to note here that *P. brasiliensis* yeast cells were not cultured *in vitro* prior RNA extraction.

### Iron depletion experiments

*P. brasiliensis* yeast cells were grown in McVeigh/Morton medium (MMcM) [52]. Iron depleted medium was supplemented with the iron chelator bathophenanthrolinedisulfonate (BPS; 50 µM; Sigma-Aldrich, Germany). *P. brasiliensis* yeast cells were incubated at 36°C with shaking at 150 rpm. Using trypan blue the number of viable cells was determined at time intervals of 2, 4, 6, 24 and 48 hours.

Protein extracts from yeast cells were prepared by inoculating 50 mL of Fava Neto's liquid medium with 10^8^ cells/mL. Cultures were incubated overnight at 36°C under gentle shaking for 16 hours. Cells were centrifuged at 5000× *g* for 5 minutes and washed 5× in MMcM media [52] containing limited iron. Control cells were incubated in MMcM supplemented with 3.5 µM Fe(NH_4_)_2_(SO_4_)_2_, for 6 and 24 hours. Cells subjected to iron starvation were incubated in MMcM medium containing 50 µM of BPS with no iron supplementation.

### Quantitative real time reverse transcription PCR (qRT-PCR) analysis

Following *P. brasiliensis* growth in iron depleted media cells were centrifuged at 1500× *g*, frozen in liquid nitrogen, and disrupted by maceration. Cells were then treated with TRIzol reagent (Invitrogen, Carlsbad, CA, USA). The manufacturer's protocol was followed to extract total RNA. The RNA was reversibly transcribed using the high capacity RNA-to-cDNA kit (Applied Biosystems, Foster City, CA, USA). We confirmed the specificity of each primer pair for the target cDNA by the visualization of a single PCR product following agarose gel electrophoresis and melting curve analysis. Primer sequences are listed in Table S4. The cDNA was quantified by qRT-PCR using a SYBR green PCR master mix (Applied Biosystems Step One Plus PCR System). qRT-PCR analysis was performed in triplicate for each cDNA sample as previously described [53]. Data were normalized to the ribosomal L34 gene (GenBank accession number EEH37825). Standard curves were generated by diluting the cDNA solution 1∶5. The standard curve method was used for relative quantification of genes [54]. Statistical comparisons were performed using the student's t test and *p*-values≤0.05 were considered statistically significant. The data were presented as fold change relation between iron deprivation and control conditions, plotted in a log2 scale of the fold change values (−Fe/Control).

### 2D-gel electrophoresis

Following *P. brasiliensis* growth in iron depleted media as described above cells were centrifuged at 1500× *g*, frozen in liquid nitrogen, and disrupted by maceration. Cells were then resuspended in a solution containing 20 mM Tris-HCl, pH 8.8, 2 mM CaCl_2_ [55]. Protein concentrations were determined using the Bradford reagent (Sigma-Aldrich) and bovine serum albumin (BSA) was utilized as a standard [56].

For each sample 300 µg of total protein were loaded onto the gel. The 2-D Clean-up Kit (GE Healthcare, Uppsala, Sweden) was used according to the manufacturer's instructions. Proteins samples were treated with 250 µL of buffer containing 7 M urea, 2 M thiourea, 130 mM 3- [(3-Cholamidopropyl)dimethylammonio]-1-propanesulfonate (CHAPS), 0.002% (w/v) dithiothreitol (DTT), ampholyte-containing buffer (IPG buffer, GE Healthcare), and trace amounts of bromophenol blue [57]. Samples were loaded onto a 13 cm Immobiline™ DryStrip gel (GE Healthcare) with a linear separation range of pH 3–11. Isoelectric focusing was conducted on a Multiphor-II electrophoresis system (GE Healthcare) using a step gradient from 500 V for 1 hour, 500–1000 V for 1 hour, 1000–8000 V over 12.5 hours, and 8000 V for 2.5 hours. The isoelectric focusing was preceded by a rehydration step at 30 V for 14 hours.

Following isoelectric focusing strips were transferred to an equilibration buffer [50 mM Tris-HCl pH 8.8, 6 M urea, 30% (v/v) glycerol, 2% (w/v) sodium dodecyl sulfate (SDS), and 0.002% (w/v) bromophenol blue] containing 18 mM of DTT for 40 minutes as previously described [58]. The strips were next transferred to a fresh equilibration buffer containing 135 mM iodoacetamide. Strips were incubated for another 40 minutes. Each IPG strip was placed on top of a polyacrylamide SDS-PAGE gel [59] and covered with agarose [0.5% (w/v) agarose in running buffer, 25 mM Tris-HCl, 192 mM glycine, 0.1% (w/v) SDS]. SDS-PAGE gels were run at 10°C for 1 hour at 150 V and increased to 250 V for 3 hours. Proteins were stained using silver or Coomassie brilliant blue (PlusOne™ Silver Staining Kit or PlusOne Coomassie Tablets PhastGel Blue R-350, GE Healthcare) according to the manufacturer's instructions.

### 2D-gel image analysis

Gel images were produced using the Image Scanner III (GE Healthcare). 2D gel spot detection, matching, and spot intensity calculations were performed using Image Master 2D Platinum v7.0 (GE Healthcare). Automated spot matching was also checked manually and corrected if needed. Spot intensity values for all protein spot matches within each analysis were calculated and normalized to the intensity of all the spots detected.

### 2D-gel statistical analysis

ANOVA statistical analysis was used to compare the differences in intensity of each matched protein spot. A comparison with a *p*-value≤0.05 was considered statistically significant. The *p*-values were calculated using the Image Master 2D Platinum v7.0 software (GE Healthcare). To check population distribution the Shapiro-Wilk test [60] was applied using the software Statistical Package for Social Science (Version 13.0, SPSS Inc, Cary, NC, USA), considering that the hypothesis of the non-normality of the sample distribution could be rejected at *p*-value≤0. 05.

### In-gel protein digestion

Protein spots were excised from the 2D-gel and diced into small pieces. Spots were next incubated with 250 µL of a solution containing 50 mM sodium thiosulfate and 15 mM potassium ferricyanide for 5 minutes. Gel pieces were washed twice with water to remove reducing agents. Next, the gel pieces were dehydrated in 100 µL of acetonitrile (ACN) and dried in a speed vacuum. The gel pieces were incubated in 50 µL of 10 mM DTT for 1 hour. The DTT solution was removed and 50 µL of 55 mM iodoacetamide was added. The gel pieces were incubated in the dark for 45 minutes. Next, the gel pieces were washed with 100 µL of 25 mM ammonium bicarbonate followed vortexing for 10 minutes. The solution covering the gel pieces was removed and gel pieces were dehydrated in 100 µL of a solution containing 25 mM ammonium bicarbonate/ACN 50% (v/v). The gel pieces were vortexed for 5 minutes and centrifuged. This step was repeated one time. Next, the gel pieces were dried and 25 µL of a 12.5 ng/ml trypsin solution was added (Sequencing Grade Modified Trypsin Promega, Madison, WI, USA). The gel pieces were placed on ice for 10 minutes. The solution covering the gels was removed and 25 µL of 25 mM ammonium bicarbonate was added. The gel pieces were incubated at 37°C for 16 hours. Following digestion the solution covering the gel pieces was transferred to a clean tube. Next, 50 µL of a solution containing 50% (v/v) ACN and 5% (v/v) trifluoroacetic acid acid (TFA) was added to the gel pieces. The gel pieces were then vortexed for 10 minutes and sonicated for 3 minutes. Next, the solution covering the gel pieces was combined with the original aqueous extraction. The samples were dried in a speed vacuum and peptides were dissolved in 10 µL of water for MS analysis [3].

### Mass Spectrometry analysis

Two microliters of each peptide sample were deposited on a matrix assisted laser desorption/ionization time-of-flight mass spectrometry (MALDI-TOF MS) target plate and dried at room temperature. Next, the peptide mixtures were covered with 2 µL of MALDI matrix solution (10 mg/mL alphacyano-4-hydroxycinammic acid in 50% (v/v) ACN, 5% (v/v) TFA). ZipTips were used to concentrate samples containing low amounts of digested protein (Milipore, Bedford, MA, USA). Dried peptides solutions were analyzed by MS. MALDI quadrupole time-of-flight (Q-TOF) MS, and MALDI-TOF-TOF MS (Synapt, Waters , Manchester, UK and Reflex IV, Bruker Daltonics, Karlsruhe, Germany, respectively). Mass spectra were collected and processed using X-TOF (Bruker Daltonics) and Mass Lynx (Waters). MS data were searched against the NCBI non redundant database and matched with their corresponding proteins sequences using MASCOT (http://www.matrixscience.com). The mass tolerance was set at 100 ppm for each MASCOT search. Each search was restricted to fungi while allowing for one missed trypsin cleavage.

### Western blot analysis

*P. brasiliensis* protein extracts of yeast cells were probed using aconitase, formamidase, isocitrate lyase, and triosephosphate isomerase antibodies. Ten micrograms of protein samples prepared as described above were loaded onto a 12% SDS PAGE gel and separated by electrophoresis. Gels were run at 150 V for ∼2 hours. Protein bands were transferred from gels to nitrocellulose membrane at 30 V for 16 hours in buffer containing 25 mM Tris-HCl, 190 mM glicine, 20% (v/v) methanol. Gels were stained with Ponceau red staining to confirm complete protein transfer. Next, each membrane was submerged in blocking buffer [Phosphate buffered saline solution 1× (PBS; 1.4 mM KH_2_PO_4_, 8 mM Na_2_HPO_4_, 140 mM NaCl, 2.7 mM KCl; pH 7.3), 5% (w/v) non-fat dried milk, 0.1% (v/v) Tween 20] for 1–2 hours. Membranes were washed with buffer [PBS 1×, 0.1% (v/v) Tween 20] and incubated with primary antibodies for 2 hours at room temperature. Primary antibodies were used at a 1/5000 (v/v) ratio of antibody to buffer. Primary polyclonal antibodies used were anti-aconitase [61], anti-isocitrate lyase (protein molecular weight 60 KDa, unpublished), anti-formamidase [62] and anti-triosephosphate isomerase [63]. This was followed by three 15 minutes washes in blocking buffer. Membranes were incubated with the appropriate conjugated secondary antibody [anti-rabbit or anti-mouse immunoglobulin G coupled to alkaline phosphatase (Sigma-Aldrich)] in a 1/5000 (v/v) ratio. Membranes were developed with 5-bromo-4-chloro-3-indolylphosphate–nitroblue tetrazolium (BCIP-NBT).

### Enzyme activity assays

Formamidase activity was determined by measuring the amount of ammonia formation as previously described [64]. One µg of *P. brasiliensis* total protein extract prepared as described above were added to 200 µl of a 100 mM formamide substrate solution in 100 mM phosphate buffer containing 10 mM of EDTA, pH 7.4. Samples were incubated at 37°C for 30 minutes. Following the 30 minutes incubation period 400 µl of phenol-nitroprusside and 400 µl of alkaline hypochlorite (Sigma Aldrich, Co.) were added. Next, samples were incubated for 6 minutes at 50°C and the absorbance was read at 625 nm. The amount of ammonia released for each sample was determined by comparing to a standard curve. One unit (U) of formamidase specific activity was defined as the amount of enzyme required to hydrolyze 1 µmol of formamide (corresponding to the formation of 1 µmol of ammonia) per minute per mg of total protein.

Catalases activity was determined by measuring a decrease in the absorbance at 240 nm from the conversion of hydrogen peroxide (H_2_O_2_) to oxygen as previously described [65]. Catalases activity for each sample was calculated using a standard curve generated by five different concentrations of H_2_O_2_. One unit of catalase activity was defined as the amount of enzyme required to catalyze the consumption of 1 µmol of H_2_O_2_ per minute per mg of total protein.

Isocitrate lyase activity was determined by measuring the formation of glyoxylate as its phenylhydrazone derivative [66]. Glyoxylate-phenylhydrazone formation was determined by measuring the absorbance at 324 nm, using an extinction coeficient of 16.8 mM^−1^ cm^−1^, in a reaction mixture containing 2 mM threo-D,L-isocitrate (Sigma Aldrich, Co.), 2 mM MgCl_2_, 10 mM phenylhydrazine HCl (Sigma Aldrich, Co.), 2 mM dithiothreitol, and 50 mM potassium phosphate at pH 7.0. Specific activity was determined as the amount of enzyme required to form 1 µmol of glyoxylate-phenylhydrazone per minute per mg of total protein.

## Supporting Information

Figure S1 **Iron starvation does not affect the viability of** ***P. brasiliensis*** **yeast cells.** Viability of *P. brasiliensis* yeast cells incubated in MMcM medium containing 3.5 µM iron (circles) and incubation of yeasts cells in MMcM iron depleted media containing 50 µM BPS (triangles). Viability was determined using trypan blue. Error bars represent standard deviation from three biological replicates while * represents *p*≤0.05.
(TIF)

Figure S2 **Categorical representation of regulated** ***P. brasiliensis*** **proteins following iron starvation.** Identified proteins were classified according to their respective functional categories determined by MIPS. (A) Categorization of differentially expressed proteins during iron starvation. Classification of proteins with induced (B) and repressed expression (C) in iron limiting condition.
(TIF)

Table S1 **Relative fold induction of iron metabolism related genes in the time course of iron starvation using real time- RT-PCR.** ^1^Values represent the mean of each triplicate sample ± standard deviation.
(DOC)

Table S2 **Additional information about** ***P. brasiliensis*** **identified proteins with increased expression during iron starvation.** ** Spots visualized only in iron-depleted condition. ^1^ Spots numbers refers to Figure 3. ^2^ *p* values were accessed by ANOVA statistical test.
(DOC)

Table S3 **Additional information about** ***P. brasiliensis*** **identified proteins with reduced expression during iron starvation.** ** Spots visualized only in iron replete condition. ^1^ Spots numbers refers to Figure 3. ^2^ *p* values were accessed by ANOVA statistical test.
(DOC)

Table S4 **Oligonucleotide primers used in quantitative RT-PCR.**
(DOC)
