# Supplementary figures and images for: Proteomic Analysis Reveals That Iron Availability Alters the Metabolic Status of the Pathogenic Fungus Paracoccidioides brasiliensis
Source: PLoS One. 2011 Jul 28;6(7):e22810. doi: 10.1371/journal.pone.0022810 (PMC3145762; doi:10.1371/journal.pone.0022810)

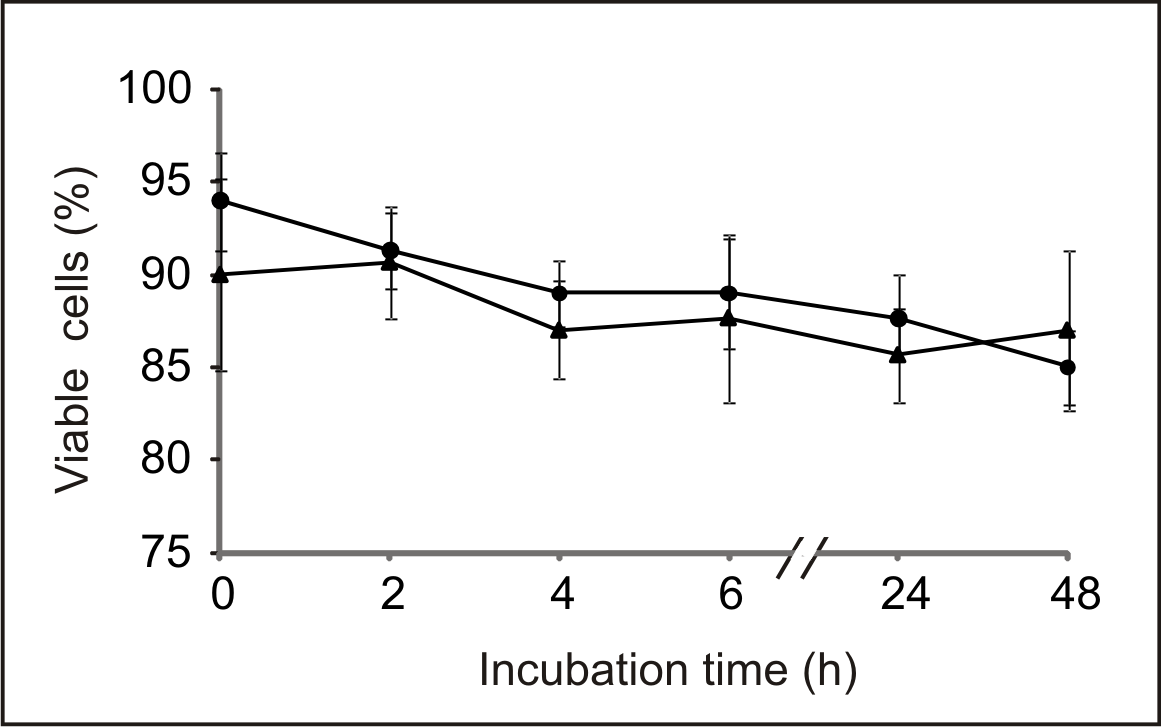

Supplement: Figure S1 — Iron starvation does not affect the viability of P. brasiliensis yeast cells. Viability of P. brasiliensis yeast cells incubated in MMcM medium containing 3.5 µM iron (circles) and incubation of yeasts cells in MMcM iron depleted media containing 50 µM BPS (triangles). Viability was determined using trypan blue. Error bars represent standard deviation from three biological replicates while * represents p≤0.05. (TIF) [file pone.0022810.s001.tif]

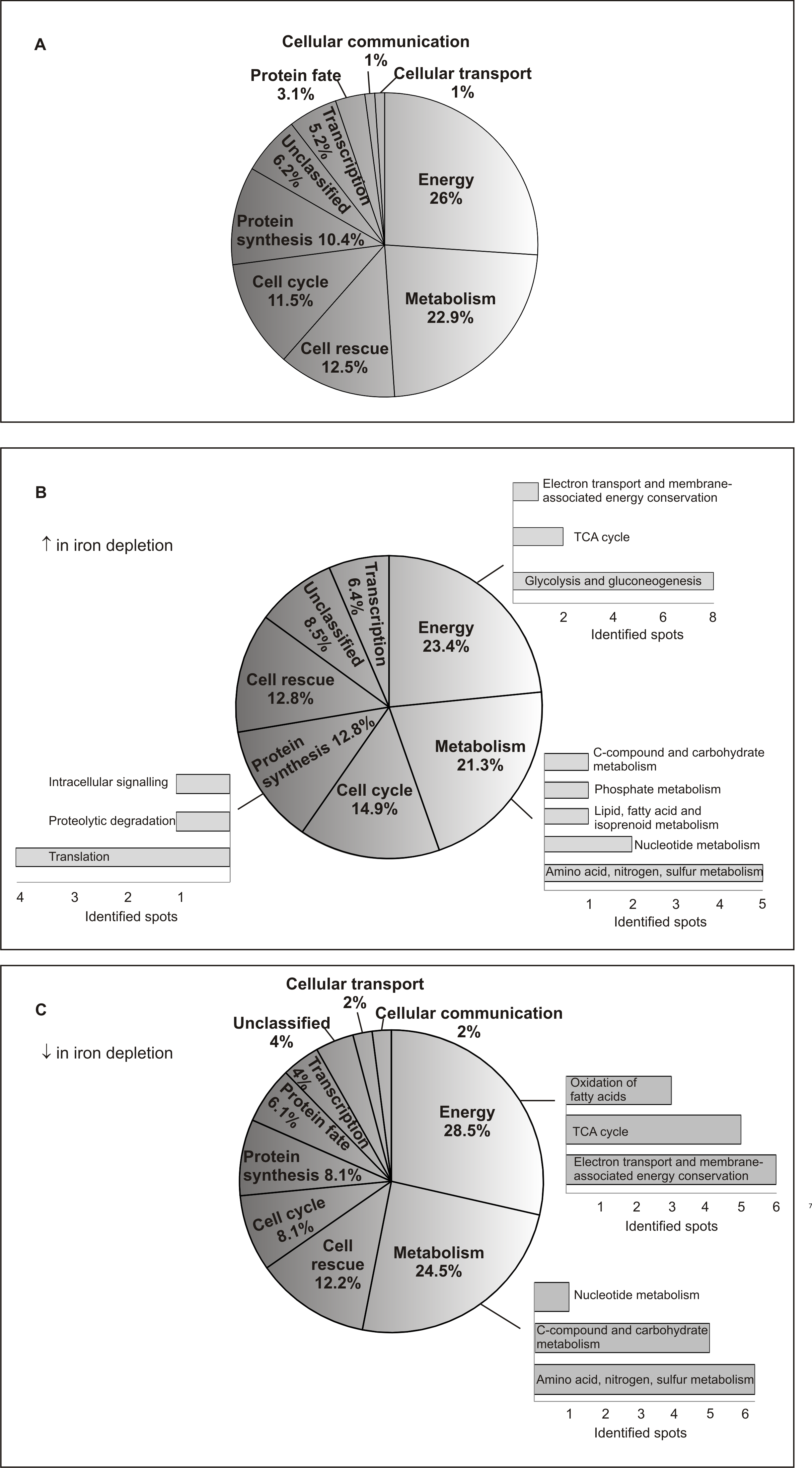

Supplement: Figure S2 — Categorical representation of regulated P. brasiliensis proteins following iron starvation. Identified proteins were classified according to their respective functional categories determined by MIPS. (A) Categorization of differentially expressed proteins during iron starvation. Classification of proteins with induced (B) and repressed expression (C) in iron limiting condition. (TIF) [file pone.0022810.s002.tif]
