# Supplementary material for: Proteomic Analysis Reveals That Iron Availability Alters the Metabolic Status of the Pathogenic Fungus Paracoccidioides brasiliensis
Source: PLoS One. 2011 Jul 28;6(7):e22810. doi: 10.1371/journal.pone.0022810 (PMC3145762; doi:10.1371/journal.pone.0022810)
Supplement: Table S1 — Relative fold induction of iron metabolism related genes in the time course of iron starvation using real time- RT-PCR. 1Values represent the mean of each triplicate sample ± standard deviation. (DOC) [file pone.0022810.s003.doc]

**Supplementary Table 1.** Relative fold induction of iron metabolism related genes in the time course of iron starvation using real time- RT-PCR.

| **Time of exposure to iron depletion^1^** | **Gene** | | |
| --- | --- | --- | --- |
|  | *hapX* | *sidA* | *sit1* |
| 5 min | 1.03 ± 0.033 | 1.07 ± 0.07 | 1.06 ± 0.016 |
| 10 min | 2.32 ± 0.025 | 2.33 ± 0.127 | 0.31 ± 0.014 |
| 30 min | 1.55 ± 0.006 | 9.66 ± 0.813 | 0.44 ± 0.024 |
| 1 h | 1.2 ± 0.036 | 1.18 ± 0.048 | 0.62 ± 0.054 |
| 3 h | 1.11 ± 0.03 | 1.29 ± 0.067 | 0.96 ± 0.068 |
| 24 h | 0.8 ± 0.07 | 1.23 ± .053 | 1.77 ± 0.080 |

^1^Values represent the mean of each triplicate sample ± standard deviation
