# Supplementary material for: Proteomic Analysis Reveals That Iron Availability Alters the Metabolic Status of the Pathogenic Fungus Paracoccidioides brasiliensis
Source: PLoS One. 2011 Jul 28;6(7):e22810. doi: 10.1371/journal.pone.0022810 (PMC3145762; doi:10.1371/journal.pone.0022810)
Supplement: Table S2 — Additional information about P. brasiliensis identified proteins with increased expression during iron starvation. ** Spots visualized only in iron-depleted condition. 1 Spots numbers refers to Figure 3. 2 p values were accessed by ANOVA statistical test. (DOC) [file pone.0022810.s004.doc]

**Supplementary Table 2.** Additional information about *P. brasiliensis* identified proteins with increased expression during iron starvation

|  |  |  | **MASCOT SCORES** | | | |  | |  |
| --- | --- | --- | --- | --- | --- | --- | --- | --- | --- |
| **Spot^1^** | **GenBank general information identifier** | **Protein identification** | **PMF** | | **MS/MS** | | | **≥ fold in iron depletion** | ***p*-value^2^** |
|  |  |  | **Score** | **Seq. cov. (%)** | **Score** | **Matched peptides** | |  |  |
|  | **METABOLISM** | | | | | | | | |
|  | **Nucleotide metabolism** | | | | | | | | |
| **1** | gi|226280544 | Adenosine kinase | 193 | 57 | 49 | 2 | | 1.3 | 3.9x10^-2^ |
| **2** | gi|226283962 | GMP synthase | 133 | 37 | - | - | | 3.6 | 1.4x10^-2^ |
|  | **C-compound and carbohydrate metabolism** | | | | | | | |  |
| **3** | gi|295664927 | ATP-citrate-lyase | 159 | 66 | 303 | 6 | | 1.2 | 1.4x10^-2^ |
|  | **Amino acid, nitrogen and sulfur metabolism** | | | | | | | | |
| **4** | gi|226280080 | 2-nitropropane dioxygenase | 163 | 56 | 107 | 3 | | ** | ** |
| **5** | gi|27368050 | Formamidase | 108 | 37 | 100 | 2 | | 1.3 | 6.6x10^-3^ |
| **6** | gi|226284927 | Hydroxyacylglutathione hydrolase | 66 | 68 | 86 | 1 | | 1.7 | 2.4x10^-2^ |
| **7** | gi|226282479 | L-threonine 3-dehydrogenase | 105 | 52 | 129 | 2 | | 1.9 | 1.2x10^-2^ |
| **8** | gi|226293104 | Spermidine synthase | 93 | 71 | 32 | 2 | | 1.6 | 3x10^-4^ |
|  | **Lipid, fatty-acid and isoprenoid metabolism** | | | | | | | | |
| **9** | gi|226279101 | Carbonic anhydrase | 90 | 45 | 104 | 2 | | 1.8 | 7x10^-4^ |
|  | **Phosphate metabolism** | | | | | | | | |
| **10** | gi|295672504 | Inorganic pyrophosphatase | 141 | 68 | 67 | 2 | | 1.3 | 4.9x10^-2^ |
|  | **ENERGY** | | | | | | | | |
|  | **Electron transport and membrane-associated energy conservation** | | | | | | | | |
| **11** | gi|226282053 | ATP synthase subunit beta | 199 | 46 | 118 | 2 | | 2.5 | 4.5x10^-3^ |
|  | **Glycolysis and gluconeogenesis** | | | | | | | | |
| **12** | gi|146762537 | Enolase | 110 | 59 | 173 | 3 | | ** | ** |
| **13** | gi|29826036 | Fructose 1,6-biphosphate aldolase | 163 | 60 | 174 | 4 | | 2.6 | 3x10^-4^ |
| **14** | PAAG_01995 | Fructose 1,6-biphosphate aldolase | 77 | 29 | - | - | | 1.4 | 3.4x10^-2^ |
| **15** | gi|226279559 | Glucokinase | 113 | 41 | 49 | 1 | | 2.8 | 6x10^-4^ |
| **16** | gi|226285327 | Phosphoglycerate kinase | 108 | 70 | 72 | 2 | | 1.6 | 4.7x10^-2^ |
| **17** | gi|295669690 | Phosphoglycerate kinase | 149 | 74 | 72 | 2 | | 1.8 | 9.1x10^-3^ |
| **18** | gi|295669690 | Phosphoglycerate kinase | 82 | 50 | 53 | 4 | | 1.2 | 1.2x10^-2^ |
| **19** | gi|295670663 | Triosephosphate isomerase (Tpi) | 185 | 51 | 87 | 3 | | 1.4 | 4x10^-2^ |
|  | **Tricarboxylic-acid pathway** | | | | | | | | |
| **20** | gi|295673931 | Pyruvate dehydrogenase protein X component | 110 | 70 | 41 | 2 | | 1.5 | 2.3x10^-2^ |
| **21** | gi|226280161 | Pyruvate dehydrogenase protein X component | 100 | 49 | 131 | 3 | | 1.4 | 2.7x10^-2^ |
|  | **CELL CYCLE AND DNA PROCESSING** | | | | | | | | |
|  | **Cell cycle** | | | | | | | | |
| **22** | gi|225683196 | Tubulin alpha-2 chain | 105 | 50 | - | - | | 1.2 | 1.4x10^-2^ |
| **23** | gi|225683196 | Tubulin alpha-2 chain | 117 | 59 | 57 | 2 | | ** | ** |
| **24** | gi|226285902 | Tubulin beta chain | 146 | 63 | 65 | 2 | | 1.8 | 3.4x10^-3^ |
| **25** | gi|154705473 | Septin-1 | 123 | 50 | 87 | 3 | | 2.0 | 4.2x10^-3^ |
| **26** | gi|226294796 | Actin | 78 | 28 | 59 | 2 | | 1.6 | 9.3x10^-3^ |
| **27** | gi|38569374 | 14-3-3-like protein 2 | 120 | 62 | 84 | 1 | | 1.7 | 3.3x10^-3^ |
| **28** | gi|226282286 | DNA damage checkpoint protein rad24 (14-3-3 protein) | 147 | 70 | 252 | 5 | | 1.4 | 2.4x10^-2^ |
|  | **TRANSCRIPTION** | | | | | | | | |
|  | **mRNA transcription** | | | | | | | | |
| **29** | gi|226280907 | mRNA binding post-transcriptional regulator (Csx1) | 92 | 33 | 44 | 2 | | 2.0 | 7x10^-4^ |
| **30** | gi|226284577 | Type 2A phosphatase activator tip41 | 90 | 60 | - | - | | 2.3 | 8x10^-4^ |
| **31** | PAAG_06891 | mRNA binding post-transcriptional regulator (Csx1) | 96 | 34 | - | - | | 2.0 | 5.4x10^-3^ |
|  | **PROTEIN SYNTHESIS** | | | | | | | | |
|  | **Translation** | | | | | | | | |
| **32** | gi|226280659 | 60S ribosomal protein L5 | 104 | 55 | - | - | | ** | ** |
| **33** | gi|226282202 | ATP-dependent RNA helicase eIF4a | 130 | 43 | 60 | 2 | | 1.3 | 4.6x10^-2^ |
| **34** | gi|226280705 | Elongation factor 2 | 103 | 21 | 110 | 3 | | ** | ** |
| **35** | gi|226283670 | Translation initiation factor eIF3 | 84 | 31 | 56 | 1 | | ** | ** |
|  | **PROTEIN FATE (folding, modification, destination)** | | | | | | | | |
|  | **Proteolytic degradation** | | | | | | | | |
| **36** | PAAG_05417 | Mitochondrial-processing peptidase subunit beta | 140 | 30 | - | - | | 1.3 | 4.1x10^-2^ |
|  | **Intracellular signaling** | | | | | | | | |
| **37** | gi|295661059 | G-protein complex beta subunit cpcb | 102 | 43 | 228 | 7 | | 3.37 | 7.2x10^-3^ |
|  | **CELL RESCUE, DEFENSE AND VIRULENCE** | | | | | | | | |
|  | **Stress response** | | | | | | | | |
| **38** | gi|60656557 | Heat shock protein 90 | 148 | 61 | 91 | 3 | | 1.4 | 4.3x10^-3^ |
| **39** | gi|226285144 | Hsp90 co-chaperone AHA1 | 150 | 61 | 48 | 2 | | 1.4 | 5x10^-2^ |
| **40** | gi|31324921 | Heat shock protein SSC1 (70 kDa) | 135 | 56 | 225 | 7 | | 1.5 | 1.5x10^-2^ |
| **41** | gi|295659116 | Hsp70-like protein | 244 | 42 | 126 | 4 | | 1.6 | 2.7x10^-3^ |
| **42** | gi|226286087 | Mitochondrial peroxiredoxin PRX1 | 197 | 86 | 97 | 3 | | 1.2 | 2.7x10^-2^ |
| **43** | gi|17980998 | Y20 protein | 92 | 46 | 82 | 2 | | 2.2 | 2.3x10^-3^ |
|  | **UNCLASSIFIED PROTEINS** | | | | | | | | |
| **44** | gi|226278304 | Conserved hypothetical protein | 103 | 41 | 40 | 1 | | 1.4 | 1.6x10^-2^ |
| **45** | gi|226286114 | Conserved hypothetical protein | 92 | 53 | 114 | 2 | | 1.6 | 8.7x10^-3^ |
| **46** | gi|226279849 | Conserved hypothetical protein | 86 | 86 | 75 | 1 | | ** | ** |
| **47** | PAAG_06617 | Conserved hypothetical protein | 113 | 31 | - | - | | 1.3 | 3.2x10^-2^ |

** Spots visualized only in iron-depleted condition

^1^ Spots numbers refers to Figure 3

^2^  *p* values were accessed by ANOVA statistical test
