# Supplementary material for: Proteomic Analysis Reveals That Iron Availability Alters the Metabolic Status of the Pathogenic Fungus Paracoccidioides brasiliensis
Source: PLoS One. 2011 Jul 28;6(7):e22810. doi: 10.1371/journal.pone.0022810 (PMC3145762; doi:10.1371/journal.pone.0022810)
Supplement: Table S3 — Additional information about P. brasiliensis identified proteins with reduced expression during iron starvation. ** Spots visualized only in iron replete condition. 1 Spots numbers refers to Figure 3. 2 p values were accessed by ANOVA statistical test. (DOC) [file pone.0022810.s005.doc]

**Supplementary Table 3.** Additional information about *P. brasiliensis* identified proteins with reduced expression during iron starvation

| **Spot^1^** | **GenBank general information identifier** |  |  | **MASCOT SCORES** | | |  |  |
| --- | --- | --- | --- | --- | --- | --- | --- | --- |
|  |  | **Protein identification** | **PMF** | | **MS/MS** | | **≤ fold in iron depletion** | ***p*-value^2^** |
|  |  |  | **Score** | **Seq. cov. (%)** | **Score** | **Matched peptides** |  |  |
|  | **METABOLISM** | | | | |  |  |  |
|  | **Nucleotide metabolism** | | | | |  |  |  |
| **48** | gi|295672652 | Bifunctional purine biosynthesis protein ADE17 | 186 | 77 | 52 | 3 | 2.0 | 1.6x10^2^ |
|  | **C-compound and carbohydrate metabolism** | | | | | |  |  |
| **49** | gi|226277934 | 2-methylcitrate synthase | 254 | 56 | 153 | 5 | 3.1 | 8.7x10^-3^ |
|  |  |  |  |  |  |  |  |  |
| **50** | PAAG_04550 | 2-methylcitrate synthase | 96 | 26 | - | - | 2.1 | 3x10^-2^ |
|  |  |  |  |  |  |  |  |  |
| **51** | gi|295666177 | Mitochondrial 2-methylisocitrate lyase | 140 | 31 | 55 | 3 | 1.5 | 4.4x10^-2^ |
| **52** | gi|116561512 | Isocitrate lyase (Icl) | 301 | 76 | 95 | 5 | 1.3 | 5x10^-2^ |
| **53** | gi|116561512 | Isocitrate lyase (Icl) | 233 | 68 | 47 | 3 | ** | ** |
|  | **Amino acid, nitrogen and sulfur metabolism** | | | | | | | |
| **54** | gi|226281118 | Pentafunctional AROM polypeptide | 189 | 33 | - | - | ** | ** |
| **55** | gi|226277980 | Acetamidase | 177 | 57 | - | - | 1.6 | 1.4x10^-2^ |
| **56** | gi|226285914 | Adenylyl-sulfate kinase | 172 | 65 | 41 | 1 | 1.9 | 2.6x10^-2^ |
| **57** | gi|225679649 | Adenylyl-sulfate kinase | 125 | 47 | 55 | 3 | 1.1 | 3.1x10^-2^ |
| **58** | gi|295663176 | Sulfate adenylyltransferase | 307 | 61 | 552 | 12 | 1.8 | 4.5x10^-2^ |
| **59** | gi|295662426 | Aspartate aminotransferase | 121 | 52 | 137 | 3 | 1.6 | 2.4x10^-2^ |
|  | **ENERGY** | | | | | |  |  |
|  | **Electron transport and membrane-associated energy conservation** | | | | | | | |
| **60** | gi|226279655 | Cytochrome c | 75 | 53 | 71 | 2 | 2.6 | 3.1x10^-2^ |
| **61** | gi|225677786 | ATP synthase gamma chain | 87 | 36 | - | - | 1.6 | 3.8x10^-2^ |
| **62** | gi|226282053 | ATP synthase subunit beta | 249 | 62 | 376 | 7 | 1.5 | 2x10^-3^ |
| **63** | gi|226279593 | ATP synthase subunit 5 | 112 | 40 | 76 | 3 | ** | ** |
| **64** | gi|226291052 | ATP synthase subunit 4 | 94 | 61 | 69 | 2 | ** | ** |
| **65** | gi|226278316 | Electron transfer flavoprotein subunit beta | 103 | 54 | 136 | 2 | ** | ** |
|  | **Tricarboxylic-acid pathway** | | | | | | |  |
| **66** | gi|226293399 | Aconitase | 172 | 33 | 211 | 4 | 1.7 | 4.1x10^-2^ |
| **67** | gi|225684009 | Aconitase | 146 | 50 | 184 | 5 | 1.7 | 1.6x10^-2^ |
| **68** | gi|225684009 | Aconitase | 139 | 43 | 55 | 2 | 1.6 | 8x10^-4^ |
| **69** | gi|226278535 | ATP-citrate synthase subunit 1 | 84 | 37 | - | - | ** | ** |
| **70** | PAAG_03330 | Dihydrolipoyl dehydrogenase | 86 | 12 | - | - | 1.3 | 2.9x10^-2^ |
|  | **Oxidation of fatty acids** | | | | | |  |  |
| **71** | gi|226278634 | Aldehyde dehydrogenase (Aldh) | 217 | 64 | 197 | 4 | 2.2 | 1.5x10^-3^ |
| **72** | gi|226286163 | 3-hydroxybutyryl CoA dehydrogenase | 165 | 64 | 101 | 3 | 1.5 | 1.4x10^-2^ |
| **73** | PAAG_06224 | Carnitine O-acetyltransferase | 89 | 17 | - | - | 2.1 | 1.4x10^-2^ |
|  | **CELL CYCLE AND DNA PROCESSING** | | | | | |  |  |
|  | **Cell cycle** |  |  |  |  |  |  |  |
| **74** | gi|154705473 | Septin-1 | 151 | 65 | 105 | 3 | ** | ** |
| **75** | gi|38569374 | 14-3-3-like protein 2 | 90 | 45 | 36 | 1 | ** | ** |
| **76** | gi|226278903 | Cell division cycle protein | 311 | 78 | 114 | 5 | 3.5 | 8.3x10^-3^ |
| **77** | PAAG_01647 | Tubulin alpha-1 chain | 103 | 32 | - | - | 1.7 | 1.5x10^-3^ |
|  | **TRANSCRIPTION** | | | | | |  |  |
|  | **mRNA transcription** | | | | | |  |  |
| **78** | gi|226277842 | Prohibitin-1 | 180 | 79 | - | - | 1.6 | 2.5x10^-2^ |
|  | **mRNA processing** | | |  |  |  |  |  |
| **79** | gi|295673504 | cwfJ domain-containing protein | 88 | 32 | - | - | 1.4 | 4x10^-2^ |
|  | **PROTEIN SYNTHESIS** | | | | | |  |  |
|  | **Translation** | | | |  |  |  |  |
| **80** | gi|226280705 | Elongation factor 2 | 283 | 63 | 244 | 6 | 3.0 | 7x10^-4^ |
| **81** | gi|226280705 | Elongation factor 2 | 189 | 61 | - | - | ** | ** |
| **82** | gi|28395450 | 40S ribosomal S12 protein | 95 | 64 | 63 | 2 | 1.7 | 4.2x10^-2^ |
| **83** | gi|226282202 | ATP-dependent RNA helicase eIF4a | 142 | 52 | 63 | 3 | ** | ** |
|  | **PROTEIN FATE (folding, modification, destination)** | | | | | |  |  |
|  | **Protein modification** | | | | | | |  |
| **84** | gi|226285231 | Ubiquitin-conjugating enzyme variant MMS2 | 126 | 63 | 153 | 5 | 1.5 | 1.4x10^-2^ |
|  | **Protein folding and stabilization** | | | | | |  |  |
| **85** | gi|34979129 | Peptidyl-prolyl cis/trans isomerase | 108 | 70 | 69 | 2 | 1.4 | 3.4x10^-2^ |
|  | **Proteolytic degradation** | | | | | |  |  |
| **86** | gi|295663176 | Dipeptidyl-peptidase | 201 | 62 | 84 | 6 | 1.7 | 2.8x10^-2^ |
|  | **CELLULAR TRANSPORT AND TRANSPORT MECHANISMS** | | | | | |  |  |
| **87** | PAAG_03137 | Vacuolar protein sorting-associated protein | 72 | 38 | - | - | 1.2 | 4.8x10^-2^ |
|  | **CELLULAR COMMUNICATION/SIGNAL TRANSDUCTION MECHANISM** | | | | | |  |  |
| **88** | gi|226285275 | Stomatin family protein | 89 | 56 | - | - | ** | ** |
|  | **CELL RESCUE, DEFENSE AND VIRULENCE** | | | | | |  |  |
|  | **Stress response** | | | | | |  |  |
| **89** | gi|60656557 | Heat shock protein 90 | 173 | 27 | 60 | 3 | 2.3 | 4.4x10^-2^ |
| **90** | gi|31324921 | Heat shock protein SSC1 (70 kDa) | 214 | 54 | 263 | 6 | 2.0 | 2.6x10^-2^ |
| **91** | gi|226278527 | 10 kDa heat shock protein, mitochondrial | 161 | 80 | 108 | 3 | 1.4 | 1.6x10^-2^ |
| **92** | gi|226282384 | 30 kDa heat shock protein | 142 | 55 | - | - | 1.6 | 8x10^-4^ |
| **93** | gi|17980998 | Y20 protein | 160 | 64 | 302 | 5 | 2.9 | 9x10^-4^ |
| **94** | gi|24528587 | Peroxissomal catalase | 71 | 32 | - | - | 2.0 | 3.5x10^-2^ |
|  | **UNCLASSIFIED PROTEINS** | | | | | |  |  |
| **95** | gi|226285365 | Conserved hypothetical protein | 135 | 64 | 194 | 4 | 1.2 | 4.7x10^-2^ |
| **96** | gi|226286445 | Conserved hypothetical protein | 154 | 52 | 37 | 2 | 2.0 | 9.7x10^-3^ |

** Spots visualized only in iron replete condition

^1^ Spots numbers refers to Figure 3

^2^  *p* values were accessed by ANOVA statistical test
