# Supplementary material for: Proteomic Analysis Reveals That Iron Availability Alters the Metabolic Status of the Pathogenic Fungus Paracoccidioides brasiliensis
Source: PLoS One. 2011 Jul 28;6(7):e22810. doi: 10.1371/journal.pone.0022810 (PMC3145762; doi:10.1371/journal.pone.0022810)
Supplement: Table S4 — Oligonucleotide primers used in quantitative RT-PCR. (DOC) [file pone.0022810.s006.doc]

**Supplementary Table 4.** Oligonucleotide primers used in quantitative RT-PCR

| **Sequence name** | **Forward primer (5´→3´)** | **Reverse primer (5´→3´)** | **Amplified product (bp)** |
| --- | --- | --- | --- |
| **Aldehyde dehydrogenase (*aldh)*** | CCTCTTACGGCCTTGCTGC | CGGACGCCCTTGATCTGAG | 170 |
| **Triosephosphate isomerase (*tpi)*** | GATGATGAGTTTGTAGCACGG | CGACCTTGCCCGTTCCGAT | 180 |
| **2-methylcitrate synthase (*2-mcs)*** | CATCTCAGTCTGCCGCTTG | CTCGTTGGCATCCAGGACA | 192 |
| **Isocitrate lyase (*icl)*** | CCGTACGAGAGGGCGACT | TCGAGATAGGTGAATTGGCAG | 196 |
| **Transcriptional factor (*hapX)*** | AGCCCAGTTCCTCCCAGAC | AAAGCAGCCTCCCGTTTGTCA | 187 |
| **Siderophore iron transporter 1 (*sit1)*** | GGCAATCATTTTCCCTGTGTG | CGCGAAGACTGCAATCAAAAG | 151 |
| **L-ornithine N^5^ –monooxygenase *(sidA)*** | GCGACGATAGCCCATTTGTC | ATTAGCAGGATTAGGATCAAGG | 155 |
| **Ribosomal protein L34** | TCTCTCCCGCGAATCCACA | AGGCGGAGAATGGGCGAG | 204 |
